# Evidence and Applicability of Stress Cardiovascular Magnetic Resonance in Detecting Coronary Artery Disease: State of the Art

**DOI:** 10.3390/jcm10153279

**Published:** 2021-07-25

**Authors:** Anna Baritussio, Alessandra Scatteia, Santo Dellegrottaglie, Chiara Bucciarelli-Ducci

**Affiliations:** 1 Department of Cardiac, Thoracic, Vascular Sciences and Public Health, Azienda Ospedale Università Padova, 35128 Padua, Italy; anna.baritussio@aopd.veneto.it; 2 Division of Cardiology, Ospedale Medico-Chirurgico Accreditato “Villa dei Fiori”, 80011 Acerra, Italy; a.scatteia@gmail.com (A.S.); santo.dellegrottaglie@mountsinai.org (S.D.); 3 Zena and Michael A, Wiener Cardiovascular Institute/Marie-Josee and Henry R. Kravis Center for Cardiovascular Health, Icahn School of Medicine at Mount Sinai, New York, NY 10029-5674, USA; 4 Royal Brompton and Harefield Hospitals, London SW3 6LR, UK; 5 Guys’s and St Thomas’ Foundation Trust and Kings College London, London SE5 9NU, UK

**Keywords:** cardiovascular magnetic resonance, stress perfusion cardiovascular magnetic resonance, coronary artery disease, myocardial ischemia, prognosis

## Abstract

Cardiovascular magnetic resonance is increasingly used in clinical practice, as it has emerged over the years as an invaluable imaging technique for diagnosis and prognosis, with clear-cut applications in managing patients with both ischemic and non-ischemic heart disease. In this review, we focus on the evidence and clinical application of stress CMR in coronary artery disease from diagnosis to prognosis.

## 1. Cardiovascular Magnetic Resonance: How Does It Work

Cardiovascular magnetic resonance (CMR) is a non-ionizing, multi-planar imaging modality that is increasingly used in clinical practice, as it is the gold standard for biventricular volumes and function assessment and is capable of accurate non-invasive myocardial tissue characterisation. 

Through excitation, inversion, and saturation pulses, CMR exploits the intrinsic properties of normal and abnormal myocardial tissue components (oedema, fibrosis etc.) and images are therefore weighted to express different pathologic processes: for example, T2-weighted images are used to detect myocardial oedema, while T1-weighted images are used to detect fibrosis and fat infiltration [1,2]. Myocardial perfusion can be assessed visually or with semi-quantitative or quantitative methods, both at rest and at stress during vasodilator (adenosine, dypiridamole, regadenoson) or dobutamine infusion, visually or with semi-quantitative or quantitative methods.

CMR uses gadolinium-based contrast agents (GBCAs) that are administered intravenously and have an extra-cellular and extra-vascular distribution so that they accumulate in expanded myocardial interstitial space. GBCAs reduce T1 times and can be detected on T1-weighted images acquired 10–20 min after administration as areas of myocardial signal enhancement (late gadolinium enhancement, LGE). Ventricular distribution pattern of LGE allows an accurate identification of myocardial damage suggesting the underlying etiopathogenesis: ischemic LGE patterns follow coronary artery distribution and the ischemic wave-front, thus initially involving the sub-endocardium and subsequently extending through the myocardial wall to become transmural in cases of prolonged myocardial ischemia; non-ischemic patterns do not follow coronary artery distribution and generally spare the sub-endocardium, having a sub-epicardial and/or mid-wall distribution [3,4,5]. 

Patients referred to CMR have to be carefully screened for potential contraindications to a CMR scan. CMR is contra-indicated in patients wearing metallic devices, such as vascular (especially cerebral) clips, ocular metallic bodies, and implanted pumps for medication infusion. Coronary stents can be scanned immediately after implantation up to 3 T, as the vessel wall provides stent stability after deployment; however, metallic artefacts do not allow the evaluation of the stent itself [6]. Most of the modern cardiac implanted devices (pace-makers and ICDs) are labelled as MR-safe or MR-conditional and can be safely scanned with caution, following the manufacturer’s instructions with regards to field strength (some devices might be safely scanned at 1.5 T but not at 3 T) and technical parameters, such as the specific absorption rate (SAR), that have to be set to reduce potential adverse effects [7]. All components of cardiac devices (i.e., generator and electrical leads) have to be MR-conditional or MR-safe in order for the CMR scan to be safely run, and it is recommended to wait six weeks after implantation or any surgical revision to allow lead stabilization [6]. A device is considered MR-unsafe in case of presence of abandoned, fractured, or epicardial leads [6]. Scanning patients with MR-conditional devices has to be planned in advance, activation and de-activation of the devices’ MR scan mode has to be organised in a timely manner, and an advanced life-support plan has to be in place. Non-MR conditional pacemakers and ICDs need to be reprogrammed by changing stimulation modality, i.e., to avoid inappropriate inhibition of pacing through inhibited or asynchronous programme, and by deactivating tachycardia detection and therapy. Inappropriate pacemaker and ICD programming may carry serious consequences not only from an arrhythmic perspective but also in terms of permanent change in device parameters (i.e. battery voltage) [8].

Systemic nephrogenic sclerosis (NSF) is the most dangerous complication potentially related to the administration of GBCAs and has been mostly reported in patients with advanced renal disease. A recent meta-analysis on 4931 patients with stage 4 and 5 chronic kidney disease (CKD) who received American College of Radiology classification group II GBCAs (gadobenate dimeglumine, gadobutrol, gadoteridol, and gadoterate meglumine), which are known to be at very low risk of NSF, found a pooled incidence of NSF of 0, with an upper bound of risk of 0.12% to 1.59% using different group II GBCAs [9]. 

Although group II GBCAs administration in CKD patients appears safer than in the past, the advent of parametric mapping, which provides a pixel-wide, colour-coded map reflecting intrinsic myocardial tissue properties, allows safe and diagnostic CMR scanning without the need of contrast agents [7]. 

## 2. Stress CMR: How to Perform It 

Pharmacological stress CMR can be performed using both vasodilator (adenosine, dypiridamole and regadenoson, and ATP) [10] and inotropic (dobutamine) agents. High event-risk pattern on stress CMR is defined by 2019 ESC guidelines on CCS as ≥2 out of 16 segments with inducible myocardial perfusion defects and ≥3 dobutamine-induced dysfunctional segments [11]. 

**Figure 1:**
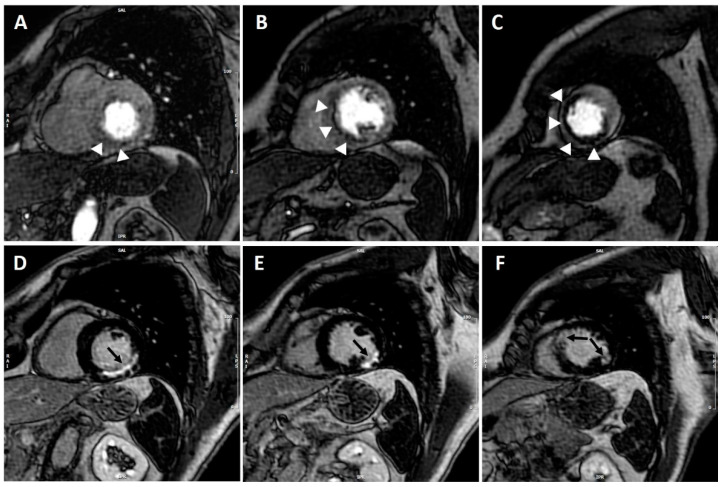
CMR adenosine-stress perfusion in a 60-year-old patient with previous history of myocardial infarction. Short axis stress perfusion images at the basal (**A**), mid-ventricular (**B**), and apical (**C**) level. There is evidence of extensive inducible perfusion defect of the basal infero-septum and inferior wall, mid-cavity septum and apical anterior, and inferior and septal walls (white arrow-heads). Corresponding LGE images (**D**–**F**) show almost transmural LGE involving the basal inferior and infero-lateral walls, the mid-cavity infero-lateral wall, and the apical antero-septum and infero-lateral wall (black arrows). CMR findings were consistent with transmural myocardial infarction in the left circumflex territory with extensive inducible ischemia in the LAD territory; coronary angiography demonstrated critical proximal LAD stenosis.

Inducible myocardial perfusion defect is visually defined on three short axis images (at the basal, mid-ventricular, and apical level, respectively) as an area of subendocardial/transmural hypo-perfusion occurring first when the contrast reaches the left ventricular myocardium (first pass) and persisting for >4 RR intervals, more than 1 pixel wide, and conforming to the distribution territory of one or more coronary arteries [7] (Figure 1). True, inducible perfusion defects have to be distinguished from artefacts, the most common being the so-called dark-rim artefact that appears as a low signal in the endocardium during early phases of myocardial perfusion study and is seen as the contrast first reaches the left ventricle but disappears once the myocardium is enhanced and is mainly a manifestation of motion artefacts. 

Although visual assessment of myocardial ischemia prevails in clinical practice, semi-quantitative and quantitative assessment of myocardial perfusion is also possible, is based on the analysis of signal intensity curves acquired during the first wash-in of contrast agent [12,13,14] and may allow quantification of absolute myocardial blood flow.

Very recent technical advances using automatically inline artificial intelligence for quantification of stress CMR perfusion mapping allow a robust quantification of myocardial blood flow (MBF) and myocardial perfusion reserve (MPR), which are strong, independent predictors of adverse cardiovascular outcomes in patients with known or suspected coronary artery disease [15].

Stress CMR also allows the identification of peri-infarct ischemia, which is generally defined as a reversible myocardial perfusion defect adjacent to and larger than infarct-related scar; although its mechanism is still debated, peri-infarct ischemia has been related to increased apoptosis; it is thought to be involved in pathological ventricular remodelling and has been shown to be associated to ventricular arrhythmias [16]. 

Vasodilator agents induce myocardial ischemia through the coronary steal phenomenon: blood flow is increased to areas supplied by patent coronaries while being simultaneously reduced to areas supplied by stenotic coronaries. Differences among vasodilator agents are reported in Table 1. Absolute contraindications to administration of vasodilator agents include acute myocardial infarction, severe hypotension (systolic blood pressure <90 mmHg), severe aortic stenosis, >II degree atrio-ventricular block (AVB), asthma, or severe obstructive pulmonary disease; caution should be used when administering vasodilator agents in patients with mild asthma, and a lower starting dose (i.e., 70 ug/Kg/min for adenosine) and beta-agonist inhalation prior to stress testing should be considered [7]. Patients should also refrain from caffeine consumption 12–24 h prior to stress testing because of its competing action on the same receptors as those of vasodilator agents. 

Termination criteria of vasodilator stress testing include persistent or symptomatic AVB, a significant drop in systolic pressure (>20 mmHg), persistent or symptomatic hypotension, and severe respiratory difficulty. 

Vasodilator stress perfusion CMR has been shown to be safe in patients with HF and reduced left ventricular ejection fraction (LVEF < 40%), with no adverse events reported in more than 1000 patients [17]. Similarly, Pezel et al. showed that vasodilator stress CMR was feasible in more than 600 patients with atrial fibrillation and suspected or stable CAD [18]. Stress perfusion CMR retains a good diagnostic quality also in patients with obesity: more than 95% of CMR scans were of diagnostic quality for perfusion in more than a thousand intermediate-risk obese patients from the SPINS registry [19].

Dobutamine stress CMR increases myocardial contractility through beta-1 stimulation, and can be used both to assess viability (at low doses) and ischemia (at high doses). Dobutamine stress CMR should not be performed in patients with serious hypertension (>220/120 mmHg), in patients with unstable angina, acute myocardial infarction, severe aortic stenosis, obstructive hypertrophic cardiomyopathy and narrow angle glaucoma, and complex cardiac arrhythmias, including uncontrolled atrial fibrillation, and in patients with myocarditis, endocarditis, or pericarditis. Termination criteria include achievement of target heart rate, a significant drop in systolic pressure (>20 mmHg), persistent symptoms, symptomatic tachycardia, and serious hypertension [20]. 

As stressor agents carry the risk of potential life-threatening arrhythmias, physicians running a stress CMR service have to be trained in advanced cardiovascular life support and have to be familiar with the crash trolley that has to be readily available [7]. ECG trace has to be continuously monitored using dedicated MR-safe equipment, and blood pressure has to be measured at regular intervals during stress testing. 

Exercise CMR has developed over the years, and it is nowadays used more frequently [21] than exercise when feasible; it is the preferred method for stress imaging according to guidelines, and exercise CMR combines the superior image quality provided by CMR with the preferred method of stress. Different types of exercise CMR are available, such as treadmill CMR, upright and supine cycle ergometer, prone exercise, and isometric handgrip. All devices used for exercise CMR need to be MR compatible so that it is possible to run the test in the scanning room close to the scanner (as with treadmill test, where the patient exercises in the scanning room outside the scanner and stress images are acquired right at peak physical stress) or directly within the scanner (as with cycle ergometer). Despite the great advantage of providing more physiological stress, exercise CMR is limited by image acquisition and quality, which is affected by motion and breathing artefacts, and by the more expensive equipment required for exercise CMR. 

## 3. Stress CMR: Guidelines Recommendations

Stress perfusion CMR has been established as a valuable diagnostic and prognostic tool and is recommended by international guidelines in the assessment of patients with coronary artery disease (CAD) [22]. 

The 2019 European Society of Cardiology (ESC) guidelines on chronic coronary syndromes (CCS) recommend the use of stress CMR as the initial test to diagnose CAD in symptomatic patients in whom obstructive CAD cannot be excluded by clinical assessment alone (class I, level of evidence B) [11]. These guidelines also recommend that the choice of the non-invasive diagnostic test be based on clinical likelihood of CAD, local expertise, and availability (class I, level of evidence C). A recent meta-analysis [23] on more than 100 patients with stable CAD using invasive coronary angiography (ICA) or ICA with fractional flow reserve (FFR) as a reference showed that functional imaging tests, such as stress CMR, are able to rule-in functionally significant CAD when pre-test probability (PTP) is ≥46–59% and rule-out when PTP is ≤34–57%. The 2014 American guidelines (American Heart Association, AHA, and American College of Cardiology, ACC) on stable coronary artery disease recommend the use of pharmacological stress CMR in patients with an intermediate to high PTP of obstructive CAD, who have an uninterpretable ECG and at least moderate physical functioning or no disabling comorbidity or are incapable of at least moderate physical functioning or have disabling comorbidity (class IIa, level of evidence B) [20,24]. Stress CMR is also considered reasonable for risk assessment in patients with stable ischemic heart disease who are able to exercise to an adequate workload but have an uninterpretable electrocardiogram (ECG) or those unable to exercise to an adequate workload regardless of interpretability of ECG (class IIa, level of evidence B) [20,24]. 

Both 2018 ESC guidelines on myocardial revascularization [25] and 2016 ESC guidelines on heart failure (HF) [26] recommend the use of stress CMR for the assessment of myocardial ischemia and viability in patients with HF and CAD, suitable for myocardial revascularization, prior to decision on myocardial revascularization (class IIb, level of evidence B). The 2014 AHA/ACC guidelines on stable ischemic heart disease recommend the use of exercise or pharmacological stress with imaging (including CMR) for risk assessment in patients with stable ischemic heart disease who are being considered for revascularization of known coronary stenosis of unclear physiological significance (class I, level of evidence B) [20,24]. 

The 2015 ESC guidelines on non-ST elevation myocardial infarction recommend the use of non-invasive imaging stress tests in patients with no recurrence of chest pain, normal ECG, and normal levels of high-sensitive troponin but suspected acute coronary syndrome before deciding on performing an invasive test (class I, level of evidence A) [27]; patients with acute chest pain and normal stress CMR have shown to have an excellent short- and mid-term prognosis. Stress CMR may be also considered to assess residual myocardial ischemia and viability in patients with ST elevation myocardial infarction, including those with multi-vessel CAD (class IIb, level of evidence C) [28]. 

The ISCHEMIA trial, a large trial on 5179 patients with moderate or severe myocardial ischemia on stress test, has shown the pivotal role of optimal medical therapy and the lack of benefit of early invasive strategies at a median follow-up of 3.2 years; however, a subgroup analysis excluding procedural infarction has suggested better outcome on longer follow-up (up to five years) in patients undergoing coronary revascularization than those receiving optimal medical therapy. Of note, as the choice of the specific test for ischemia detection was based on physician preference or local availability, only 5% of enrolled patients had ischemia detected by stress CMR as compared to nearly 50% detected by SPECT; therefore, the study is likely not powered to assess the prognostic role of stress CMR [29]. Nevertheless, cumulative evidence of the diagnostic and prognostic role of stress CMR, described in detail in the next paragraph, supports the use of this imaging technique in clinical practice. Importantly, ISCHEMIA is not a trial of imaging approaches to chest pain, and as a result, imaging (both computed tomography coronary angiography and ischemia testing) were used to set exclusion and inclusion criteria, respectively. Hence, ISCHEMIA is not helping draw any conclusion on the role of ischemia testing in patients with chest pain. This position is clearly presented by the American Society of Echocardiography, American Society of Nuclear Cardiology, and the Society for Cardiovascular Magnetic Resonance, among other scientific societies [30]. 

## 4. Stress CMR: Evidence from Literature

### 4.1. Diagnosis 

Evidence of the diagnostic role of stress perfusion CMR has significantly increased over the last two decades. In the late 2000s the multi-centre, multi-vendor MR-IMPACT trial [31], by comparing stress perfusion CMR and single photon emission computed tomography (SPECT) for the detection of CAD, demonstrated equal performance of the two techniques in the head-to-head comparison and CMR superiority in patients with multi-vessel coronary artery disease. The multi-centre, multi-vendor MR-IMPACT II trial [32] demonstrated a superior sensitivity of perfusion CMR as compared to SPECT in the detection of CAD (67% vs. 59%), as confirmed by invasive coronary angiography, but inferior specificity (61% vs. 72%). 

The CE-MARC trial recruited 752 patients with suspected angina and at least one cardiovascular risk factor undergoing adenosine stress perfusion CMR, SPECT, and coronary angiography as reference standard: CMR showed high diagnostic accuracy for the detection of CAD and proved to be superior to SPECT both in single and multi-vessel disease [33]. A sub-analysis of the CE-MARC trial showed that the maximum sensitivity for the detection of significant CAD by CMR was provided by the use of all four CMR study components (left ventricular function, myocardial inducible ischemia, myocardial viability by LGE, and coronary magnetic resonance angiography, MRA)(86.5%), which also represented the optimum strategy to rule-out significant CAD; on the other hand, specificity of LGE was the highest (95.8%), and the LGE component alone was the best to rule-in CAD [34]. Adding coronary MRA did not provide additional benefit when compared to the combination of perfusion/function/LGE. 

More recently, the MR-INFORM trial showed that stress perfusion CMR is associated with a lower incidence of coronary revascularization than invasively assessed fractional flow reserve (FFR) (35.7% of patients vs. 45.0%, respectively) and that CMR is non-inferior to FFR with regards to the incidence of major adverse cardiac events [35].

The advent of computed tomography-derived FFR (FFR_CT_), a novel, non-invasive functional test, has recently prompted the comparison with stress perfusion CMR for prediction of coronary revascularization in patients with stable angina and one or more coronary stenosis (≥50%) on CT. A recent study from Rønnow Sand NP et al. showed that, although FFR_CT_ had higher sensitivity than perfusion CMR (97% vs. 47%, respectively) to identify patients needing coronary revascularization, stress perfusion CMR had higher specificity (88% vs. 42% by FFR_CT_) and positive predictive value (67% vs. 47%) [36]. Semiquantitative CT myocardial perfusion imaging has shown similar performance to adenosine or regadenoson stress CMR in the detection of CAD as compared to the reference standard of invasive coronary angiogram and SPECT. A meta-analysis on 2048 patients also showed a higher pooled diagnostic accuracy for stress CMR, CT, and positron emission tomography, as compared to SPECT and echocardiography, in the detection of haemodynamically significant CAD [37] both at the vessel and patient level, with similar negative likelihood ratios for perfusion imaging. 

Additionally, stress perfusion CMR has been shown to be a cost-effective test in different international studies. Stress perfusion CMR has been compared to immediate coronary angiography with selected FFR, SPECT, coronary CT with FFR_CT_, and no imaging [38] in a United States cohort of symptomatic patients with 30% prevalence of obstructive CAD; stress perfusion CMR has proved to be a cost-effective tool to detect CAD prior to invasive coronary angiography. Similar findings have been reported in a United Kingdom (U.K.) study that showed that stress perfusion CMR in stable CAD is cost-effective as compared to myocardial perfusion scintigraphy and to UK National Institute for Health and Care Excellence (NICE) guidelines-guided care overall and across all CAD pre-test likelihood subgroups [39].

Besides the described role of stress CMR over other imaging modalities in the detection of myocardial ischemia, it has to be noted that CMR carries other clinically-relevant advantages: it does not use ionizing radiations (as opposed to SPECT, CT, and invasive coronary angiography), it has higher contrast-to-noise ratio than CT when assessing myocardial perfusion, and has good temporal resolution but lower spatial resolution as compared to CT [37,40].

### 4.2. Prognosis 

Given the high diagnostic yield of stress CMR and considering that it is a very comprehensive examination able to provide information on ventricular function, stress myocardial perfusion, and viability within a single study, much effort has recently been made to provide reliable evidence on its prognostic value [35,41]. The multi-centre Stress CMR Perfusion Imaging in the United States (SPINS) study demonstrated the value of stress CMR for predicting the occurrence of death and/or myocardial infarction as well as the cost-effectiveness of this approach [42]. In this retrospective study, consecutive patients from 13 centres across the U.S., presenting with a chest pain syndrome, were referred for stress CMR and were followed for a target period of four years. The authors found that patients with no CMR evidence of ischemia or LGE experienced low annualized rates of primary outcome, defined as cardiovascular death or non-fatal myocardial infarction. On the other hand, patients with ischemia and LGE had a four-fold higher annual rate of primary outcome as well as a 10-fold higher incidence of coronary revascularization during the first year after CMR. Similar findings had been previously shown by Vincenti et al. in 2017 [43]: they demonstrated not only that patients without ischemia had excellent outcomes but also that a worse prognosis was related to ischemia extent in terms of number of involved myocardial segments (>1.5 ischemic segments) and that ischemia was a stronger predictor of primary and secondary endpoints than LV ejection fraction (EF) and scar burden as assessed by LGE. In a large cohort of 6389 patients with known or suspected stable ischemic heart disease who underwent vasodilator stress CMR, Marcos-Garces et al. highlighted that the risk of all-cause mortality increased in parallel with the extension of the ischemic burden, whereas the opposite tendency was observed in patients who underwent CMR-guided revascularization [44]. In addition to this, stress-CMR has been proven to help patients’ risk classification besides conventional cardiovascular risk factors [45]. Recently, a retrospective multi-centre cohort study included 1698 patients with two or more coronary risk factors but no history of CAD, who were referred to stress CMR imaging for suspected myocardial ischemia. Guideline-based categories of risk, in terms of pre-test likelihood of CAD, were applied [11]; outcomes were cardiovascular death, non-fatal myocardial infarction, hospitalization for heart failure or unstable angina, and late, unplanned coronary artery bypass graft surgery. The results showed that stress CMR imaging provided risk reclassification in 60.3% of patients in the intermediate pre-test risk category, supporting the potential value of stress CMR for clinical decision making, especially in patients in the intermediate risk category [46]. In another study, Indorkar et al. showed that a lower coronary flow reserve, derived by stress CMR, was significantly associated with higher risk of major adverse cardiac events even after adjustment for clinical and conventional imaging risk factors (e.g., LVEF, LGE size, and ischemia extent) [47]. 

Finally, many studies tried to estimate the prognostic role of stress CMR in specific populations. In a sub-analysis of the SPINS study, focused on patients with reduced LVEF (<50%), the authors showed that the presence of ischemia, LGE, or both was associated with higher event rates and that both of them were independent predictors of primary (cardiovascular death or non-fatal myocardial infarction) and secondary outcomes (a composite of cardiovascular death, nonfatal myocardial infarction, hospitalization for unstable angina or congestive heart failure, and unplanned late coronary artery bypass graft surgery) [48]. These findings were also confirmed by Pezel et al. in another study on 950 patients with heart failure and LVEF < 40% [17]. Among the specific populations, the prognostic role of stress CMR has been already assessed in obese patients (BMI > 30 kg/m^2^), in patients with atrial fibrillation and suspected or stable coronary artery disease, as well as in elderly patients (>75 years) with suspected coronary artery disease [18,49,50] and female patients. In all these studies, stress CMR has been shown to be feasible and of prognostic relevance. 

## 5. Stress CMR during COVID-19 Pandemic

Severe acute respiratory syndrome coronavirus 2 (SARS-CoV-2) has caused a pandemic of respiratory disease since late 2019 (referred to as COVID-19 pandemic), determining millions of deaths to date [51]. Clinical practice has been heavily affected, and in- and out-patient clinics have needed extensive transformation in order to provide the best of care and, at the same time, limit the risk of disease transmission. The European Association of Cardiovascular Imaging has provided recommendations on cardiac imaging during the COVID-19 pandemic that highlight the importance of performing cardiac imaging only in those cases where it is believed to substantially change patients’ management or be life-saving. The experts report a very limited role of stress imaging techniques during COVID-19 pandemic and state that any stress test should be avoided during acute infection in order to limit the risk of infection for the involved personnel and of contamination for the equipment; coronary CT angiography is to be preferred instead in patients investigated for chronic coronary syndromes [52]. The Society for Cardiovascular Magnetic Resonance (SCMR) has also produced guidance documents on the use of CMR, including stress CMR, in the convalescent patients with COVID-19 in which signs and symptoms of cardiac involvement persisted [53,54,55]. 

## 6. Conclusions

Stress CMR is a safe and feasible test for assessing patients with known or suspected coronary artery disease, and this holds true also in specific categories of subjects, such as women, obese patients and patients with severe left ventricular dysfunction, atrial fibrillation, left bundle branch block. Currently, vast amounts of clinical data support the evidence on diagnostic and prognostic role as well as the cost-effectiveness of CMR-based clinical strategies. Unfortunately, availability of dedicated CMR scanners and of experienced operators may still be limited on a local base. On the other hand, increasing recognition of the key role of CMR in the assessment of many non-ischemic cardiac conditions, in many cases, is acting as a practical stimulus for clinical cardiologists to consider stress CMR as a valuable alternative to other tests for non-invasive ischemia detection. 

## Figures and Tables

**Table 1:** Vasodilator agents used for stress CMR.

	Adenosine	Dypiridamole	Regadenoson
Potency	Less potent		10 times more potent
Receptor Selectivity	Non-selective A_2_		Selective A_2A_
Administration	Weight-based 4–6 min infusion	Weight-based 6 min infusion	Non-weight based, single dose bolus
Dose	140 ug/Kg/min	0.56–0.84 mg/Kg	400 ug
Duration of Infusion	4–6 min	4–6 min	10 s bolus
Time to Peak Plasma Concentration	30 s	minutes	33 s
Half-life	<10 s	11 h	30 min
Duration of Action	6 s	30 min	2.3 min
HR	Smaller increase		Faster and greater increase
Adverse Effects	More bronchocostriction, AVB		Less bronchocostriction, AVB

HR, heart rate; AVB, atrio-ventricular block.

## Data Availability

Not applicable.
